# Biofilm Disrupting Technology for Orthopedic Implants: What’s on the Horizon?

**DOI:** 10.3389/fmed.2014.00022

**Published:** 2014-08-15

**Authors:** Alexander Connaughton, Abby Childs, Stefan Dylewski, Vani J. Sabesan

**Affiliations:** ^1^Michigan State University College of Human Medicine, Grand Rapids, MI, USA; ^2^Western Michigan University Homer Stryker MD School of Medicine, Kalamazoo, MI, USA

**Keywords:** biofilms, orthopedics, joint replacements, novel technology, periprosthetic fractures, infectious disease medicine

## Abstract

The use of orthopedic implants in joints has revolutionized the treatment of patients with many debilitating chronic musculoskeletal diseases such as osteoarthritis. However, the introduction of foreign material into the human body predisposes the body to infection. The treatment of these infections has become very complicated since the orthopedic implants serve as a surface for multiple species of bacteria to grow at a time into a resistant biofilm layer. This biofilm layer serves as a protectant for the bacterial colonies on the implant making them more resistant and difficult to eradicate when using standard antibiotic treatment. In some cases, the use of antibiotics alone has even made the bacteria more resistant to treatment. Thus, there has been surge in the creation of non-antibiotic anti-biofilm agents to help disrupt the biofilms on the orthopedic implants to help eliminate the infections. In this study, we discuss infections of orthopedic implants in the shoulder then we review the main categories of anti-biofilm agents that have been used for the treatment of infections on orthopedic implants. Then, we introduce some of the newer biofilm disrupting technology that has been studied in the past few years that may advance the treatment options for orthopedic implants in the future.

Implants have become an indispensable part of orthopedic medicine. The use of orthopedic implants has revolutionized the treatment of patients with debilitating diseases like osteoarthritis and bone fractures. However, the compromise of the skin barrier during surgical procedures and the introduction of foreign material during joint replacement may predispose the body to infection, including biofilm creating bacteria.

A biofilm is an assemblage of surface-associated microbial cells that is enclosed in an extracellular polymeric substance (EPS) matrix (1). Biofilms are enhanced by the ability of bacteria to attach to an orthopedic implant through their surface structures such as pili, fimbriae, flagella, and glycocalyx (2). Additionally, adherence of microorganisms to the surface of the implant may involve non-specific factors like surface tension, hydrophobicity, and electrostatic forces (1). In general, any surface is susceptible to biofilm growth, but rougher and more hydrophobic surfaces will accumulate biofilms more rapidly (3). These bacterial biofilms can grow to reach thicknesses of 100 mm (4). The depletion of nutrients that can easily occur at the implant surface results in a slow growing stationary state that can render the biofilm up to 1000 times more resistant to most antimicrobial agents when compared to the single bacteria cell (4). Eradicating these complicated slow growing biofilms with antibiotic therapy alone becomes extremely difficult. Prosthetic joint biofilms can be mono- or polymicrobial and are frequently caused by *Staphylococcus aureus*, coagulase-negative Staphylococci, beta-hemolytic Streptococci, and aerobic Gram negative rods (including *Pseudomonas aeruginosa*) (5) Moreover, the timeline of the biofilm formation has proven to be critical to its susceptibility to antibiotics (6). In other words, within the first week of formation, a biofilm might be susceptible to tobramycin and piperacillin and subsequently become resistant (6). Treatment with only antibiotics can increase the resistance of these infections and there have even been cases where they have stimulated the growth of the biofilms (7, 8).

The development of these complicated infections after orthopedic implant surgery is a major problem. There are about one million joint replacements, hip, knee, and shoulder (9, 10), performed each year in the United States, which is expected to exceed four million by 2030 (11, 12). Infection rates for hip, knee, and shoulder arthroplasty at most centers are reported to be <2%, with most of these occurring in the first 2 years post-op (4, 8, 11, 13). One and two-stage revision can serve as definitive treatment and success rates have been documented in case-series to be 80 and 90%, respectively (14–17). Additionally, aggressive debridement and retention of the implant is an option in select cases of early-onset infection (30 days after implant) with no evidence of loosening or sinus tract formation (18). This strategy in early-onset infection may be curative in combination with long-term antibiotics in up to 71% of cases (19–21). Finally, resection without reimplantation is a last resort (16). However, the cost and morbidity of periprosthetic joint infections and revision joint arthroplasty can be exorbitant (22). Conservative estimates suggest that each prosthetic joint infection may cost $50,000 or more (23), and the total economic burden of these prosthetic joint infections will only continue to increase with the number of total joint arthroplasties performed every year (13, 22). That is why it is imperative that we continue to pursue other methods besides antibiotics to help prevent and treat biofilm-forming infections. We will review the different types of technology that have been developed to deter biofilm formation and introduce some of the newer interventions being researched to combat these infections.

Modification of the orthopedic implants is one of the first aspects looked at in preventing these infections. The type of alloy used in implants has been the focus of many research studies. When testing the ability of some of the more common bacteria in implant infections such as the *Staphylococcus* spp., it was found that the bacteria had decreased ability to adhere and create a biofilm layer on titanium than stainless steel or polymethylmethacrylate (24). This can be explained by the ability of titanium to keep the bacteria dispersed on the implant surface making the bacteria more susceptible to antibiotics (25). This antimicrobial effect is one reason why titanium alloy has become one of the more popular alloy used in orthopedic implants. A recent study looked at innovations using titanium alloy investigating vanadium free titanium alloys. They found this specialized alloy had decreased bacterial adherence and biofilm formation than titanium that contains vanadium (26).

The field of anti-biofilm agents has been increasing dramatically and there is an exhaustive list of agents in each category of this field as described in Campoccia et al. (27). Agents that have bacteria repelling and anti-adhesive surfaces target the factors that enhance adhesion of the bacteria to the orthopedic implant that are ubiquitous to the common infecting bacteria (27). These anti-biofilm agents utilize hydrophilic, highly hydrated, and anionic surfaces that are typically most difficult for bacteria to adhere to (27). Polyethylene oxide is an example of this category that can be applied to a synthetic surface such as an orthopedic implant to prevent bacterial attachment and biofilm development (28). While most anti-biofilm surface repelling agents use hydrophilic properties, there is also a recent example of an agent that uses a hydrophobic polycationic surface on titanium and stainless steel implants. These were found to inhibit biofilm formation and also enhance bone healing in an infectious environment (29).

Another category of anti-biofilm agents are intrinsically bioactive materials that are non-antibiotic compounds with innate antibacterial properties in their structure. Examples in this category are metals such as silver and copper. The antibacterial properties of the metals come from their corrosive properties that result in ion release that can disrupt essential processes of the bacteria such as those in the respiratory chain (27). These metals can be integrated onto the surface of an orthopedic implant to prevent adhesion of the bacteria.

There are many examples of bioactive antibacterial coatings that are placed on top of an implant surface that have active antibacterial properties. One example of this is when the implant surface is cross-linked with a bioactive molecule such as human b-defensin-3. Recent studies on the antimicrobial peptide human b-defensin-3 show that it has the capacity to reduce the number of bacterial colonies that accumulate on titanium surfaces and can be effective against resistant organisms such as methicillin-resistant *Staphylococcus epidermidis* (MRSE) and methicillin-resistant *Staphylococcus aureus* (MRSA) (30, 31). Another type of bioactive antibacterial coating releases nitric oxide that can combine with superoxide to produce peroxynitrite, which has very cytotoxic actions against bacteria (27). Polymer coatings-containing diazeniumdiolates are an example of a nitric-oxide releasing coating (27, 32). Along the same line are reactive oxygen species-releasing coatings such as polycaprolactone incorporating calcium peroxide (27). Studies have shown that bursts of calcium peroxide can be bactericidal while only having short term toxic effects to surrounding host tissues (33). There are also photoactivated bioactive biomaterials that can be activated at certain UV wavelength to exhibit their bactericidal effects such as the anatase TiO_2_, which is activated at the 385 nm UV wavelength (27).

Another category is nanostructured biomaterials that contain compounds such as silver or chitosan that have antibacterial properties (27). The nanostructures can also be used to alter the surface functionality (solubility, surface charge, and other properties) of the implant making it more difficult for bacteria to attach (27, 34).

Research is being focused on the use of vaccines against bacteria that commonly infect implants. These vaccines contain polysaccharide or protein from the bacterial surface. When they are administered, the body develops immunoglobins against these bacterial proteins or polysaccharides to prevent the biofilm formation (34). Most attempts at developing a vaccine against *Staphylococcus aureus* have been largely unsuccessful due to the adaptability and phenotypic variation exhibited by this bacteria (35). One example that showed promise was the StaphVAX against *Staphylococcus aureus* capsular polysaccharides. StaphVAX successfully made it to phase III clinical trials, but was withdrawn when the vaccine’s effectiveness in producing the immunoglobins against the bacteria decreased to <30% in the year (36). A recently developed quadrivalent vaccine against *Staphylococcus aureus* (targeting glucosaminidase, an ABC transporter lipoprotein, a conserved hypothetical protein, and a conserved lipoprotein) was able to clear 87.5% of the bacterial biofilm infections in combination with antibiotics compared to 22% in those just given with the vaccine (37). This shows the importance of combining vaccine and antibiotic treatment with this therapy if it becomes successful clinically in the future.

The use of bacteriophages, which are viruses that infect and destroy bacteria, have recently been explored for their effects to remove biofilms in orthopedic implants. One study found that bacteriophages enhanced the effects of antibiotics in eliminating orthopedic implant infections of MRSA and *Pseudomonas aeruginosa* in rat models (38).

Bioactive enzymes lyse certain elements of the biofilm aggregating on the orthopedic implant resulting in the destruction of the biofilm. For example, dispersin B, which lyses polymers or Proteinase K, which lyses proteins of the biofilm structure both of which make the bacteria more susceptible to antibiotic treatment (27). A number of cytotoxic agents have also been found to be successful in removing biofilms from implant surfaces. In a recent review, the most successful cytotoxic agent found to eliminate biofilms on titanium surfaces was citric acid (39). While there is limited literature on these agents, they show promise and more studies are needed to fully understand their efficacy.

Another new field being researched is the use of electrical stimulation on orthopedic implant surfaces. Studies have shown that when an electrical current was applied to a stainless steel implant infected with *Staphylococcus aureus* and *Staphylococcus epidermis*, the current was able to enhance detachment of the biofilm (40–42). One of these studies tested the use of an electric current *in vivo* with rabbit models that had an external power source that was attached to an infected stainless steel implants that provided a current. They found this model to be successful in reducing the biofilm load (41). Applying an electrical current to infected implants in combination with antibiotic therapy could be favorable treatment algorithm in the future so that patients could avoid having to go through major single-stage revision or two-stage reimplantation procedures.

Another potential innovation applied pulsed electromagnetic fields to stainless steel pegs infected with biofilms of *Staphylococcus epidermis* in combination with antibiotics. This study reported a 50% decrease in the minimum biofilm inhibitory concentration needed for gentamicin; however, this effect was not seen with vancomycin (43).

A new surge in research has been in the use of laser-generated shockwaves, which use mechanical energy to break up biofilms (35). One study found that around 97.9% of *Pseudomonas aeruginosa* biofilms on nitinol stents could be removed with just 4–10 s of applying the laser (44). Laser-generated shockwaves were able to break up the biofilm layer into bacteria in its single-celled planktonic form that can be more easily treated with antibiotics (44).

We can no longer rely solely on antibiotics and surgery to treat these infections because of the increased patient costs and morbidity. In addition, the current treatment algorithms are becoming increasingly less effective with more virulent organisms and bacterial resistance. The application of various technologies and different disciplines can advance the field of biofilm disrupting technology. Innovative biofilm-disrupting-treatments are at the forefront of medical research as scientists continue to look for new technology to combat the complicated bacteria that infect implants.

## Conflict of Interest Statement

The authors declare that the research was conducted in the absence of any commercial or financial relationships that could be construed as a potential conflict of interest.

## References

[1] Donlan RM. Biofilms: microbial life on surfaces. Emerg Infect Dis (2002) 8(9):881–90. 10.3201/eid0809.020063 12194761 PMC2732559

[2] Renner LD Weibel DB. Physicochemical regulation of biofilm formation. MRS Bull (2011) 36(5):347–55. 10.1557/mrs.2011.65 22125358 PMC3224470

[3] Ribeiro M Monteiro FJ Ferraz MP. Infection of orthopedic implants with emphasis on bacterial adhesion process and techniques used in studying bacterial-material interactions. Biomatter (2012) 2(4):176–94. 10.4161/biom.22905 23507884 PMC3568104

[4] Zoubos AB Galanakos SP Soucacos PN. Orthopedics and biofilm – what do we know? A review. Med Sci Monit (2012) 18(6):RA89–96. 10.12659/MSM.882893 22648264 PMC3560733

[5] Berbari EF Hanssen AD Duffy MC Steckelberg JM Ilstrup DM Harmsen WS Risk factors for prosthetic joint infection: case-control study. Clin Infect Dis (1998) 27(5):1247–54. 10.1086/514991 9827278

[6] Anwar H Strap JL Chen K Costerton JW. Dynamic interactions of biofilms of mucoid Pseudomonas aeruginosa with tobramycin and piperacillin. Antimicrob Agents Chemother (1992) 36(6):1208–14. 10.1128/AAC.36.6.1208 1416820 PMC190319

[7] Campoccia D Montanaro L Speziale P Arciola CR. Antibiotic-loaded biomaterials and the risks for the spread of antibiotic resistance following their prophylactic and therapeutic clinical use. Biomaterials (2010) 31(25):6363–77. 10.1016/j.biomaterials.2010.05.005 20542556

[8] Cargill JS Upton M. Low concentrations of vancomycin stimulate biofilm formation in some clinical isolates of *Staphylococcus epidermidis*. J Clin Pathol (2009) 62(12):1112–6. 10.1136/jcp.2009.069021 19640858

[9] Gilson M Gossec L Mariette X Gherissi D Guyot MH Berthelot JM NIH consensus conference: total hip replacement. NIH consensus development panel on total hip replacement. JAMA (1995) 273(24):1950–6. 10.1001/jama.273.24.1950 7783307

[10] Kurtz S Ong K Lau E Mowat F Halpern M. Projections of primary and revision hip and knee arthroplasty in the United States from 2005 to 2030. J Bone Joint Surg Am (2007) 89(4):780–5. 10.2106/JBJS.F.00222 17403800

[11] Bohsali KI Wirth MA Rockwood CA Complications of total shoulder arthroplasty. J Bone Joint Surg Am (2006) 88(10):2279–92 10.2106/JBJS.F.00125 17015609

[12] Lee B Joint Replacement in U.S. Exceed 1 Million a Year. Pittsburgh, PA: Scripps Howard News Service (2013). Available from: http://www.post-gazette.com/news/health/2013/03/04/Joit-replacements-in-U-S-exceed-1-million-a-year/stories/201303040218

[13] Piper KE Jacobson MJ Cofield RH Sperling JW Sanchez-Sotelo J Osmon DR Microbiologic diagnosis of prosthetic shoulder infection by use of implant sonication. J Clin Microbiol (2009) 47(6):1878–84. 10.1128/JCM.01686-08 19261785 PMC2691098

[14] Biring GS Kostamo T Garbuz DS Masri BA Duncan CP. Two-stage revision arthroplasty of the hip for infection using an interim articulated Prostalac hip spacer: a 10- to 15-year follow-up study. J Bone Joint Surg Br (2009) 91(11):1431–7. 10.1302/0301-620X.91B11.22026 19880885

[15] Brandt CM Duffy MC Berbari EF Hanssen AD Steckelberg JM Osmon DR *Staphylococcus aureus* prosthetic joint infection treated with prosthesis removal and delayed reimplantation arthroplasty. Mayo Clin Proc (1999) 74(6):553–8 10.4065/74.6.553 10377928

[16] Sia IG Berbari EF Karchmer AW. Prosthetic joint infections. Infect Dis Clin North Am (2005) 19(4):885–914. 10.1016/j.idc.2005.07.010 16297738

[17] Zeller V Lhotellier L Marmor S Leclerc P Krain A Graff W One-stage exchange arthroplasty for chronic periprosthetic hip infection: results of a large prospective cohort study. J Bone Joint Surg Am (2014) 96(1):e1. 10.2106/JBJS.L.01451 24382729

[18] Osmon DR Berbari EF Berendt AR Lew D Zimmerli W Steckelberg JM Diagnosis and management of prosthetic joint infection: clinical practice guidelines by the Infectious Diseases Society of America. Clin Infect Dis (2013) 56(1):e1–25 10.1093/cid/cis803 23223583

[19] Byren I Bejon P Atkins BL Angus B Masters S McLardy-Smith P One hundred and twelve infected arthroplasties treated with ‘DAIR’ (debridement, antibiotics and implant retention): antibiotic duration and outcome. J Antimicrob Chemother (2009) 63(6):1264–71. 10.1093/jac/dkp107 19336454 PMC2680346

[20] El HelouOC Berbari EF Lahr BD Eckel-Passow JE Razonable RR Sia IG Efficacy and safety of rifampin containing regimen for staphylococcal prosthetic joint infections treated with debridement and retention. Eur J Clin Microbiol Infect Dis (2010) 29(8):961–7. 10.1007/s10096-010-0952-9 20505968

[21] Zimmerli W Widmer AF Blatter M Frei R Ochsner PE. Role of rifampin for treatment of orthopedic implant-related staphylococcal infections: a randomized controlled trial. Foreign-Body Infection (FBI) Study Group. JAMA (1998) 279(19):1537–41. 10.1001/jama.279.19.1537 9605897

[22] Poultsides LA Liaropoulos LL Malizos KN The socioeconomic impact of musculoskeletal infections. J Bone Joint Surg Am (2010) 92(11):e13 10.2106/JBJS.I.01131 20810849

[23] Sculco TP The economic impact of infected joint arthroplasty. Orthopedics (1995) 18(9):871–3. 8570494

[24] Gad GFM Aziz AAA Ibrahem RA In-vitro adhesion of *Staphylococcus* spp. to certain orthopedic biomaterials and expression of adhesion genes. J Appl Pharm Sci (2012) 2(6):145–9 10.7324/JAPS.2012.2634

[25] Lauderdale KJ Malone CL Boles BR Morcuende J Horswill AR. Biofilm dispersal of community-associated methicillin-resistant *Staphylococcus aureus* on orthopedic implant material. J Orthop Res (2010) 28(1):55–61. 10.1002/jor.20943 19610092

[26] Walkowiak-Przybyło M Klimek L Okrój W Jakubowski W Chwiłka M Czajka A Adhesion, activation, and aggregation of blood platelets and biofilm formation on the surfaces of titanium alloys Ti6Al4V and Ti6Al7Nb. J Biomed Mater Res A (2012) 100(3):768–75. 10.1002/jbm.a.34006 22238248

[27] Campoccia D Montanaro L Arciola CR. A review of the biomaterials technologies for infection-resistant surfaces. Biomaterials (2013) 34(34):8533–54. 10.1016/j.biomaterials.2013.07.089 23953781

[28] Roosjen A Kaper HJ van der Mei HC Norde W Busscher HJ. Inhibition of adhesion of yeasts and bacteria by poly(ethylene oxide)-brushes on glass in a parallel plate flow chamber. Microbiology (2003) 149(Pt 11):3239–46. 10.1099/mic.0.26519-0 14600236

[29] Schaer TP Stewart S Hsu BB Klibanov AM. Hydrophobic polycationic coatings that inhibit biofilms and support bone healing during infection. Biomaterials (2012) 33(5):1245–54. 10.1016/j.biomaterials.2011.10.038 22082621

[30] Huang Q Yu HJ Liu GD Huang XK Zhang LY Zhou YG Comparison of the effects of human β-defensin 3, vancomycin, and clindamycin on *Staphylococcus aureus* biofilm formation. Orthopedics (2012) 35(1):e53–60. 10.3928/01477447-20111122-11 22229614

[31] Zhu C Tan H Cheng T Shen H Shao J Guo Y Human β-defensin 3 inhibits antibiotic-resistant *Staphylococcus* biofilm formation. J Surg Res (2013) 183(1):204–13. 10.1016/j.jss.2012.11.048 23273885

[32] Nablo BJ Prichard HL Butler RD Klitzman B Schoenfisch MH. Inhibition of implant-associated infections via nitric oxide release. Biomaterials (2005) 26(34):6984–90. 10.1016/j.biomaterials.2005.05.017 15978663

[33] Wang J Zhu Y Bawa HK Ng G Wu Y Libera M Oxygen-generating nanofiber cell scaffolds with antimicrobial properties. ACS Appl Mater Interfaces (2011) 3(1):67–73. 10.1021/am100862h 21155527

[34] Romanò CL Toscano M Romanò D Drago L. Antibiofilm agents and implant-related infections in orthopaedics: where are we? J Chemother (2013) 25(2):67–80. 10.1179/1973947812Y.0000000045 23684354

[35] Hansen EN Zmistowski B Parvizi J. Periprosthetic joint infection: what is on the horizon? Int J Artif Organs (2012) 35(10):935–50. 10.5301/ijao.5000145 23371923

[36] Shinefield H Black S Fattom A Horwith G Rasgon S Ordonez J Use of a *Staphylococcus aureus* conjugate vaccine in patients receiving hemodialysis. N Engl J Med (2002) 346(7):491–6. 10.1056/NEJMoa011297 11844850

[37] Brady RA O’May GA Leid JG Prior ML Costerton JW Shirtliff ME. Resolution of *Staphylococcus aureus* biofilm infection using vaccination and antibiotic treatment. Infect Immun (2011) 79(4):1797–803. 10.1128/IAI.00451-10 21220484 PMC3067561

[38] Yilmaz C Colak M Yilmaz BC Ersoz G Kutateladze M Gozlugol M Bacteriophage therapy in implant-related infections: an experimental study. J Bone Joint Surg Am (2013) 95(2):117–25 10.2106/JBJS.K.01135 23324958

[39] Ntrouka VI Slot DE Louropoulou A Van der Weijden F. The effect of chemotherapeutic agents on contaminated titanium surfaces: a systematic review. Clin Oral Implants Res (2011) 22(7):681–90. 10.1111/j.1600-0501.2010.02037.x 21198891

[40] Ercan B Kummer KM Tarquinio KM Webster TJ. Decreased *Staphylococcus aureus* biofilm growth on anodized nanotubular titanium and the effect of electrical stimulation. Acta Biomater (2011) 7(7):3003–12. 10.1016/j.actbio.2011.04.002 21515421

[41] Del PozoJL Rouse MS Euba G Kang CI Mandrekar JN Steckelberg JM The electricidal effect is active in an experimental model of *Staphylococcus epidermidis* chronic foreign body osteomyelitis. Antimicrob Agents Chemother (2009) 53(10):4064–8. 10.1128/AAC.00432-09 19651912 PMC2764171

[42] van der Borden AJ van der Mei HC Busscher HJ. Electric block current induced detachment from surgical stainless steel and decreased viability of *Staphylococcus epidermidis*. Biomaterials (2005) 26(33):6731–5. 10.1016/j.biomaterials.2004.04.052 15979141

[43] Pickering SA Bayston R Scammell BE. Electromagnetic augmentation of antibiotic efficacy in infection of orthopaedic implants. J Bone Joint Surg Br (2003) 85(4):588–93. 10.1302/0301-620X.85B4.12644 12793569

[44] Kizhner V Krespi YP Hall-Stoodley L Stoodley P. Laser-generated shockwave for clearing medical device biofilms. Photomed Laser Surg (2011) 29(4):277–82. 10.1089/pho.2010.2788 21182450

